# Analysis of desert traffic accidents: A retrospective study

**DOI:** 10.5339/qmj.2024.65

**Published:** 2024-12-26

**Authors:** Haris Iftikhar, Suha Turkmen, Aftab Mohammad Azad, Zain Bhutta, Melih Imamoglu, Serdar Karakullukcu, Amr Mohammed Elmoheen, Jassim Mohammad, Hany Attia Zaki Mahmoud, Ahmed Ibrahim Sheashaa, Guillaume Alinier

**Affiliations:** 1 Department of Emergency Medicine, Hamad General Hospital, Doha, Qatar; 2 Department of Emergency Medicine, Qatar University, Doha, Qatar *Email: STurkmen@hamad.qa; 3 Department of Emergency Medicine, School of Medicine, Karadeniz Technical University, Trabzon, Turkey; 4 Department of Public Health, School of Medicine, Karadeniz Technical University, Trabzon, Turkey; 5 Hamad Medical Corporation Ambulance Service, Doha, Qatar; 6 Weill Cornell Medicine-Qatar, Education City, Doha, Qatar; 7 School of Health and Social Work, University of Hertfordshire, College Lane, Hatfield, United Kingdom; 8 Faculty of Health and Life Sciences, Coach Lane Campus, North Umbria University, Newcastle upon Tyne, United Kingdom

**Keywords:** Desert car accidents, road traffic accidents, motor vehicle crashes, ATV, trauma, injuries, Qatar

## Abstract

**Introduction:**

Motor vehicle accidents (MVAs) are a leading cause of mortality and morbidity worldwide. There is limited literature on the injuries resulting from desert off-road driving accidents. This study aims to delineate the attributes of desert MVA and associated injuries as observed in Qatar.

**Methods:**

This is a Qatar-based retrospective descriptive multicenter study from electronic medical records (EMRs) between 2016 and 2022. Patients were evaluated based on demographic characteristics, type of injury and vehicle involved, injury locations, injury severity, treatments applied, length of hospital stay, number and outcome of surgeries, disability on discharge, and mortality.

**Results:**

Findings reveal a rising trend in accidents from October to March, peaking between 4:00 p.m. and 8:00 p.m. The patients were predominantly male under 40 years of age, not adhering to personal protective measures, and accidents often involved all-terrain vehicles (ATVs) and sport utility vehicles (SUVs). Blunt trauma emerged as the primary injury type, with orthopedic injuries being the most frequent. Most patients did not undergo surgery and had an average hospital stay of 2.9 days. A 1.5% mortality rate and 6.7% disability rate were observed.

**Conclusion:**

This study fills a critical gap in understanding desert traffic accidents in a Gulf Cooperation Council country. This study underscores the need for targeted interventions and public awareness campaigns tailored to the unique challenges of desert driving.

## 1. Introduction

Road traffic accidents (RTAs), also called motor vehicle crashes (MVCs) or motor vehicle accidents (MVAs), are a leading cause of mortality and morbidity, especially in the young population.^1,2^ 1.3 million people worldwide die because of RTA. About 50 million people suffer from non-fatal RTA, but the number of disabled individuals cannot be underestimated.^3^ Traffic accidents on highways are analyzed, and many studies are carried out to prevent these accidents and reduce possible injuries and associated costs.^4^ The medical literature also reveals the characteristics of these accidents, identifies common types of injuries, and suggests appropriate treatments. There are warning signs in critical areas on highways where traffic rules exist, and camera or police roadside observation and control points are established to ensure compliance with road safety regulations.^5^ The desert environment, with no highways, warning signs, or strict and enforceable rules, creates a different setting for MVA.

In recent years, the deserts of the Arabian Peninsula and Qatar have become popular destinations for people to engage in extreme sports and leisure activities. Qatar is transforming into a central modern sports and tourism hub after hosting significant events such as the 2006 Asian Games and the 2022 FIFA World Cup.^6,7^ Prevention of trauma-related deaths remains a major challenge in Qatar, with 23% of deaths attributed to trauma, making it one of Qatar’s most important health issues. Injuries most often occur due to MVA in Qatar^8^ and the increasing popularity of vehicle use in desert regions when the weather is pleasant has also led to various accidents and injuries. In the desert, where there are no traffic regulations, precautions, or controls in place, several factors contribute to the occurrence of traffic accidents. Among these factors are the use of vehicles by individuals unfamiliar with the desert terrain and lacking experience in driving under challenging conditions, the use of standard vehicles designed for conventional roads in the desert, and driving in desert without adopting adequate safety measures. There is also the overconfident use of 4 × 4 vehicles at high speed for adrenaline rush seekers practicing dune bashing without following basic safety measures such as keeping a safe distance from other vehicles, all passengers wearing a seatbelt, and reducing speed when there is poor visibility. Altered visibility may be caused by sandstorm or dust, poor lighting, the desert creating visual illusions or mirages, or dunes hiding obstacles such as other vehicles or sudden drops in the terrain. Due to the relative commonality of MVA in the desert in Qatar, the national Ambulance Service has invested in appropriate resources to rapidly respond by air or land to accidents in the desert.^8^ During the peak season of people going into the desert, Hamad Medical Corporation (HMC) even sets up a temporary clinic at “Sealine,” near the main entry point of the “inland sea” desert, and has multiple nearby ambulance spoke stations.^9,10^

There is limited information on the injuries resulting from desert off-road driving accidents, sports, and tourism-related activities in desert environments available in the medical literature. Investigating these injuries and their results could provide essential insight into the extent and characteristics of these incidents, as desert trip-related activities are an integral part of Middle Eastern culture and tourism. It could help make new informed recommendations in terms of safety measures to be adopted.

Alongside Qatar’s growing population, we believe that traffic accidents in desert regions are increasing gradually, displaying distinctive features compared to accidents happening on conventional roads. In this retrospective study, our primary objective is to delineate the attributes of MVAs and associated injuries that occurred in the desert in Qatar over the period of 2016–2022 based on the records held by Hamad Medical Corporation Ambulance Service (HMCAS) and HMC hospitals.

## 2. Methods

This study is a retrospective descriptive multicenter cross-sectional study. Ethical approval was obtained from HMC Medical Research Centre (MRC 01-22-571). A detailed chart review was conducted using the HMC’s electronic medical records (EMRs) between 2016 and 2022. Qatar’s HMC Ambulance Service’s EMR has been initially searched for all patients transported from desert locations in Qatar. Only MVA-related trauma patients in the deserts were included. The types of MVA that caused the injury were determined. Patients were evaluated based on demographic characteristics, vehicle involved, type of injury and injury locations, injury severity, treatments applied, hospital length of stay, number and outcome of surgeries, disability on discharge, and mortality. Subsequent hospital records of the patients were reviewed for complications. Patients were divided into age groups. Vehicle types were categorized as follows in our analysis: Quad bikes, buggies, and motorized 3-wheelers were classified as all-terrain vehicles (ATVs). All four-wheel-drive or all-wheel-drive cars were categorized as sport utility vehicles (SUVs), while some were simply categorized as “cars” based on the information entered on the patients’ EMR.

SPSS 24.0 statistical package program was used in the analysis of the data. Descriptive statistics of evaluation results included numbers and percentages for categorical variables, mean, standard deviation, median, and first and third quartiles for numerical variables. The Chi-square test was used to compare the categorical variables of independent groups. The statistical alpha significance level was accepted as *p* < 0.05.

## 3. Results

In this retrospective study, we examined a total of 3,262 patients who were involved in traffic accidents in the desert between 2016 and 2022. However, for various reasons, including incomplete patient identification information in the HMCAS EMR, patients declining to go to the Emergency Department by ambulance, and the transfer of children to a pediatric trauma center, only 1,496 patients could be included in our detailed analysis. Additionally, it is important to note that some data variables were missing for certain patients included in the study.

**Figure 1.**
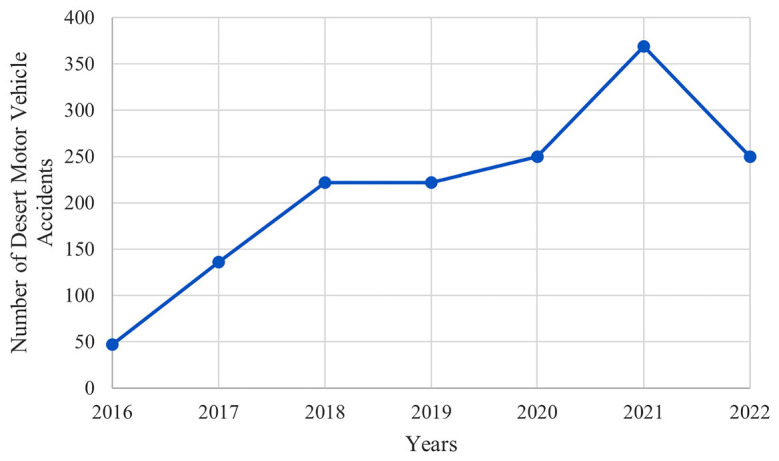
Number of yearly Motor Vehicle Accidents (MVA) that occurred in the desert between 2016 and 2022 resulting in injuries.

**Figure 2.**
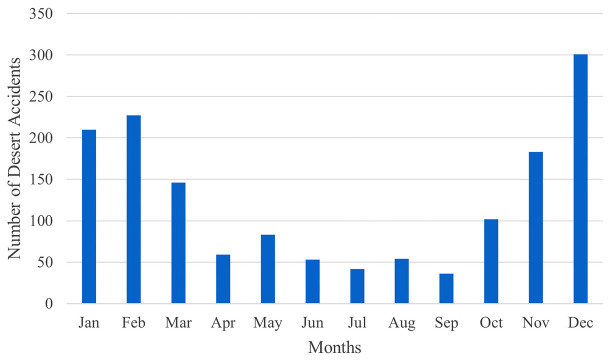
Number of monthly Motor Vehicle Accidents that occurred in the desert between 2016 and 2022 resulting in injuries.

**Figure 3.**
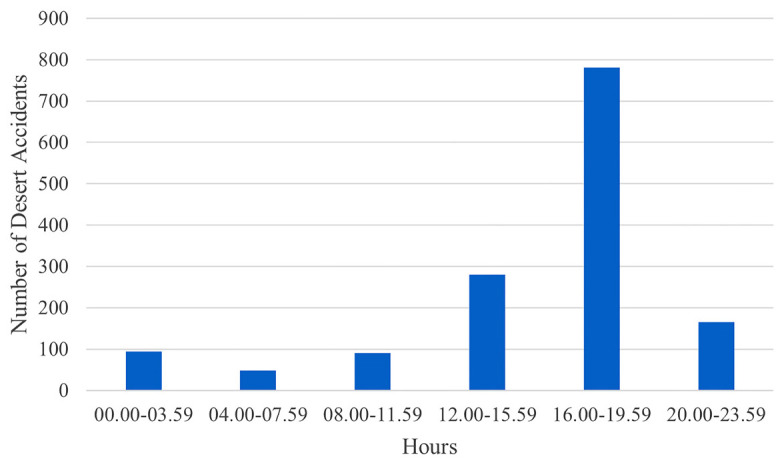
Time of the day when Motor Vehicle Accidents resulting in injuries occurred in the desert between 2016 and 2022.

Although there was no serious change in the population during the years included in the study, when we examined desert accidents over the years, we saw that the number of accidents was gradually increasing (Figure 1). In addition, when analyzing by months of the year, we notice that the period between October and March, spanning six months, sees a higher frequency of accidents (Figure 2). Regarding the time of day, most accidents occurred between 4:00 p.m. and 8:00 p.m. (Figure 3).

The mean age of the patients was 24.6 years old, with the majority of them being under 40 years of age. Desert traffic accidents affected mostly men. Most of the accident victims were expatriate residents, followed by local nationals. Only a small number of patients were intoxicated due to alcohol consumption, and a similar number were pregnant. The demographic characteristics of the patients are shown in Table 1. It was observed that most accidents involved ATVs (67.4%), followed by SUVs (27.4%). Most of these vehicles or patients lacked protective measures or equipment, and rollovers were the most common accident type (Table 2).

Blunt trauma was the most common type of injury (Supplementary Table 1). Most patients did not require surgery during their hospital stay, and only 18.1% of those involved in the accidents were operated on (Table 3). On average, patients stayed in the hospital for 2.9 days. Unfortunately, many patients were lost to follow-up after leaving the hospital. Although most cases resulted in minor injuries, there was a 1.5% mortality rate and a 6.7% disability rate based on the sample for which we had data continuity between the pre-hospital and hospital EMR systems. Tragically, 22 patients died in desert traffic accidents, most suffering traumatic cardiac arrest on scene, although limited data is available concerning them. Despite these challenges, the overall outcome for most patients was positive.

Table 4 shows that 11.7% of patients had a compromised airway, with 2.7% experiencing direct airway and tracheobronchial injuries. Blunt neck trauma was seen in 9.1% of patients, with 3.7% having C-spine injuries. Thoracic injuries were noted in 13.9% of patients, including thoracic fractures (5.6%), lung contusions (3.2%), hemothorax (0.9%), emphysema (1.7%), pneumothorax (1.5%), and flail chest (0.1%). Abdominal and pelvic injuries were present in 8.3% of patients. A traumatic brain injury (TBI) was observed in 15.1% of patients, with only 3.4% having a Glasgow Coma Scale (GCS) score of less than 15 upon arrival of the Ambulance Service crew on scene (Supplementary Table 2).

Table 5 reveals that the most frequent injuries in desert MVAs are orthopedic injuries, accounting for 35.6% of cases. Among them, 22.4% of patients had closed fractures, and only 2.1% had open fractures. Oral and maxillofacial or ear, nose, and throat (OMF/ENT) injuries were observed in 8.1% of patients, while 2.2% had ophthalmology injuries. Most patients with orthopedic injuries were under 40 years old (Supplementary Table 3).

## 4. Discussion

In recent years, the growing population across the region and desert activities such as off-road driving and dune bashing have become popular in the Gulf states,^11^ and it has been accompanied by increased MVA in desert areas.^12^

The findings of our first nationwide study of desert MVA in Qatar revealed several important insights into the patterns and consequences of these accidents. There is minimal data on these accidents in the literature. Our study provided comprehensive data on the number of patients exposed to desert traffic accidents, their demographic characteristics (such as age, gender, and residency status), types of vehicles involved in the accidents, types of injuries sustained, the need for surgery, hospital length of stay, disability rate, specific injury patterns (e.g., compromised airway, C-spine injuries, thoracic injuries, abdominal and pelvic injuries, TBI, and orthopedic injuries), and mortality rate.

With this study, we aimed to highlight the number of desert traffic accidents, analyze the outcomes of these accidents, and inform official authorities and the public about this issue and the measures that can be taken to prevent and manage desert traffic accidents.

One of the important findings of our study is that the number of traffic accidents occurring in the desert is increasing despite no significant change in the number of people living in Qatar (Figure 1). While the Qatar National Traffic Safety Strategy, implemented since 2013, has effectively reduced fatalities from road traffic accidents, the substantial occurrence of accidents in the desert regions can be attributed mainly to the absence of comprehensive traffic monitoring and enforcement of safety measures in these areas.^13^ The lack of traffic laws and enforcement can lead drivers to take risks and drive more dangerously, resulting in accidents. This observation highlights the need for accident prevention campaigns, safety measures, and infrastructure in desert areas to prevent such accidents, in addition to what is being proposed or already implemented on the road.^4^

Another notable finding is the seasonal variation in accident numbers. Especially during the autumn and winter months, when an increase in desert MVAs has been observed, with December being the month with the highest number of accidents. This is because of the popularity of the winter camping season which is associated with increased vehicle traffic in the desert. This higher level of activity being linked to a higher number of MVA is a not surprising outcome and has also been reported in a study conducted in the United Arab Emirates.^14^

The region’s traffic density and weather conditions can influence the seasonal variation of MVAs. Timmermans and colleagues analyzed road traffic accidents in Qatar between 2010 and 2016 and determined that accidents resulting in serious and fatal injuries occurred most frequently in the autumn and winter. They suggested this was due to the higher occurrence of dense fog and severe winds during these seasons.^13^ This could also be linked to the exodus of many residents traveling abroad during the hot summer months, resulting in a drop in the country’s population and hence reduced traffic on the roads.

In a study by Bellos and colleagues in Greece between 2011 and 2015, it was demonstrated that the increase in traffic density during touristic seasons and the necessity for tourists to drive in unfamiliar traffic conditions led to an increase in traffic accidents during periods of heightened tourist activity.^15^

Although our study found a higher incidence of injuries among non-Qataris, we think this is related to the much higher number of non-Qataris than Qataris living in the country. Approximately 15% of the population in Qatar consists of Qataris, the male–female ratio is approximately 3/1, and the population mean age is around 30 years old.^16^ The increase in desert accidents along with the increase in desert travel activities in these months can be associated with the factors mentioned in previous studies, such as the traffic density in these months, including adverse weather conditions, and the aggravation of the difficulties of driving in desert environments.

Upon analyzing the timing of accidents, we observed that most accidents occurred between 4:00 p.m. and 8:00 p.m., which is after normal working hours and hence a time when people go out to socialize. Notably, these hours also coincide with sunset in Qatar, particularly during the winter season, possibly leading to reduced visibility. Furthermore, due to the relatively milder temperatures during these hours, many activities in desert areas tend to occur, leading to increased traffic.

Therefore, it is crucial to prioritize traffic safety strategies in the desert during these crash-prone times. Drivers must be aware of the unique challenges of driving in desert environments, such as limited visibility due to sandstorms and sand dunes or extreme temperatures that can impact vehicle performance. Best practices for driving safely in the desert should be promoted. This could include information on preparing vehicles for extreme conditions and tips for avoiding common hazards like stunt driving and not allowing for a sufficient safety distance when driving near other vehicles. A comprehensive, multi-faceted approach is required by focusing on driver and passenger education, infrastructure, and emergency response to reduce the risk of accidents and improve safety during the camping seasons.

There are several similarities between accidents in the desert and those on highways, where males and young adults are reported to be prone to accidents.^17,18^ Within our study, we observed a predominant occurrence of orthopedic injuries, encompassing injuries to the thoracic and lumbosacral spinal regions, followed by traumatic brain injuries, thoracic injuries, airway trauma, blunt neck trauma, abdominal/pelvic trauma, and OMF/ENT injuries (Table 4). The most frequently injured body regions are the extremities, followed by maxillofacial injuries, head/neck injuries, and chest injuries in road traffic accidents as well.^19^ In a study carried out in Qatar, an analysis was conducted on injuries associated with ATVs and revealed that the body regions most affected by injuries were the chest, followed by the upper extremities and the head.^20^ On the other hand, like our study, a retrospective study of ATV-related accidents in the desert found that the most common injuries involved the skin and musculoskeletal systems, including fractures. The most common sites of injury were the extremities.^21^ Another study on motorcycle desert racing revealed similar findings, with fractures of the extremities being the most common injuries.^22^

The most frequent reasons for TBI are falls and RTAs. The likelihood of fatality varies depending on the injury’s severity and the patient’s age. The case fatality rate for TBI generally ranges from 0.9% to 7.6%, while the rate for severe TBI is between 29% and 55%.^23^ In major accidents, victims often experience multiple injuries, with thoracic injuries occurring in about 50% of cases. Thoracic trauma is also the third leading cause of death in multi-trauma patients, and it can result in unfavorable short-term outcomes, accounting for up to 25% of trauma-related deaths.^24^ Road traffic injuries were the leading cause of injury in both in-hospital and out-of-hospital traumatic cardiac arrest cases in Qatar. Over 90% of traumatic cardiac arrest patients experienced blunt injuries, which may be specific to the region.^25^ Similarly, blunt trauma was the most common injury type observed in desert MVA patients in the present study. It has also been reported that patients who suffered out-of-hospital traumatic cardiac arrest had a higher frequency of severe polytrauma injuries, leading to immediate or earlier onset of cardiac arrest.^25^ Traumatic aortic dissection is an exceedingly uncommon occurrence, with most patients dying on scene. In our study, only two patients experienced this type of injury.

The incidence of airway and tracheobronchial injuries poses a challenging task for management due to their associated high fatality rate. Presently, acute traumatic airway injuries are estimated to occur in the range of 0.5% to 2% of cases involving MVA and pedestrian injuries.^26,27^ In our study, we observed that airway-related issues manifested in 11.7% of patients involved in the desert MVA, with 2.7% experiencing direct airway or tracheobronchial injury. These figures are consistent with those reported for highway accidents.^26,27^ Moreover, 8% of patients exhibited a compromised airway due to oromaxillofacial injuries (OMFs), while others presented with airway obstructions caused by blood, vomit, secretions, or foreign objects (Table 4).

Our patients were typically less intoxicated than those in other studies.^19,21^ Only a small number of pregnant women were involved, and there were no cases of morbidity or mortality among them (Table 1). These numbers are less than those stated in the literature.^28^ The most common type of vehicle involved in desert MVAs was the ATV. The literature review on the mechanism of injury during ATV accidents showed that these vehicles could be very challenging to operate, even with smaller engines and specific engine recommendations based on age. The data indicate that the driver usually had difficulty controlling the vehicle, resulting in rollovers, falls, and collisions. Passengers were mainly injured in rollovers.^29^ Additionally, there is a notable deficiency in the utilization of protective equipment such as helmets, with only 25.8% of the individuals in our study employing them (Table 2). A similar investigation conducted between 2010 and 2018 into ATV-related injuries in Qatar exhibited a remarkably low helmet usage rate at 3.6%, indicating a pervasive insufficiency in using protective gear.^29^ In this context, it is understood that while there are some safety measures in place at vehicle rental locations, but compliance is not enforced as they are not governed by strict regulations. However, there is no regulation regarding speed limits, the age of drivers, or the duration of holding a driver’s license. It is evident that there are no enforced controls or precautions when people travel in the desert with their own vehicles. Implementing such measures is believed to have the potential to significantly reduce the risk of serious injuries.

The differences between highway and desert traffic accidents relate to various factors including the terrain conditions, traffic volume, and driver behavior. Desert terrains may include sand dunes, gravel, and uneven surfaces, which can be challenging, especially for drivers unfamiliar with the environment. Qatar’s natural terrain is mostly constituted of calcareous sandy loam, covered with rock debris, with a few areas covered by sand dunes.^30^ We do not believe that the “soft” sand contributed to reducing the severity of the injuries or the mortality rate as it does not provide any real cushioning when vehicle passengers are ejected then crushed by their own vehicle, or when they collide with hard surfaces inside their vehicle. In remote desert areas, traffic volume may be relatively low, leading to less congestion but longer response times for emergency services in the event of an accident. Early detection of highway accidents allows for a quick response from pre-hospital emergency services, but detecting accidents in the desert can be challenging if there is no one able to call 999. Ambulance transports from desert areas had longer scene times and total prehospital times. Reducing access time of the emergency medical response team relies on good communication and coordination of all parties involved, including the dispatch center who will determine where the closest response unit is.^31^ In rural and desert areas, helicopter emergency medical service (HEMS) utilization was more frequent, possibly due to the severity of injuries and ground ambulance transportation challenges due to geographical distance and access issues. This is especially true for recreational-related injuries in the desert.^8^ Waiting for help in the desert can increase the risk of hypovolemia, heat stroke, and death.^32^ Understanding these differences is essential for implementing targeted measures to prevent accidents and improve desert driving safety.

## 5. Limitations

We encountered challenges in calculating our patients’ injury severity scores (ISS), which would have been valuable for comparative analysis with prior studies. Additionally, our dataset lacked comprehensive information on traumatic cardiac arrests due to the unfortunate circumstances of many patients passing away at the accident scene or a lack of identifiable information in the Ambulance Service data precluding retrospective chart tracing. These patients were included, but details of their injuries could not be described in our analysis. The retrospective analysis of disability data presented difficulties, as many disability scoring systems lack validation for retrospective patient cohorts and chart reviews. One common issue we were confronted with was missing data. Additionally, we were not able to report any information regarding if the accidents involved rented or private vehicles and whether the patients were drivers or passengers, as this is not systematically documented by the paramedic crews treating patients. It is essential to acknowledge that limitations are a natural part of any retrospective study. In that respect, we encourage clinicians and paramedics to properly document what they observe on the scene and the information they gather from bystanders or relatives, as it can help improve patient care.^33^

## 6. Conclusion

This MVA study has identified a concerning trend of gradually increasing traffic accidents in the desert region throughout the study duration. Notably, the seasonal fluctuations and peak accident hours parallel those seen in typical road traffic incidents, with a prevalence during the winter and autumn months, particularly between 4 p.m. and 8 p.m. These observations emphasize the significance of targeted traffic safety interventions during these time frames and the necessity for public awareness campaigns and infrastructure improvements tailored to address the distinctive challenges associated with desert driving and providing emergency medical care in remote desert areas.

The study findings revealed demographic patterns similar to highway accidents, with a disproportionate impact on young males, but it is also representative of the country’s general population demographics. Accidents involving primarily ATVs, often resulting in rollovers, emphasize the importance of safety regulations for desert vehicle operation. The most prevalent types of injuries resulting from these accidents were orthopedic injuries, followed by traumatic brain injuries, thoracic trauma, airway trauma, blunt neck trauma, abdominal/pelvic trauma, and OMF/ENT injuries. Despite minor injuries being common, it is notable that the mortality rate stood at 1.5% and the disability rate at 6.7%. The overall favorable outcomes for most patients upon discharge emphasize the critical importance of timely and comprehensive response and medical care.

This study fills a critical gap in understanding desert traffic accidents in Qatar. The data serve as a valuable resource for policymakers, healthcare professionals, and researchers striving to enhance desert driving safety and reduce the impact of off-road traffic accidents.

## Acknowledgment

The team wishes to thank Mr. Mohammed Hardan from the Hamad Medical Corporation Ambulance Service Business Intelligence Unit for his support in providing the data specifically related to accidents that occurred off-road. The authors want to acknowledge Dr. Ali Waseem, Dr. Ayisha Ameen, and Dr. Omair Malik for providing help in data collection for this study.

## Conflict of Interest Statement

The author(s) of this work have nothing to disclose.

## MRC Approval

Ethical approval was obtained from the Hamad Medical Corporation’s (HMC) Institutional Review Board (MRC-01-22-571).

## Authors’ Contributions

HI, ST, ZB, GA: Conceptualization; Writing—original draft preparation; Data curation; Formal analysis; Funding acquisition; Methodology; Project administration. MI, SK: Formal analysis; Writing—review & editing. AA, AE, JM, HM, AS: Conceptualization; Resources; Supervision; Validation; Writing—review & editing.

## Authorship Declaration

All authors agree with the content of the manuscript.

## Supplementary Material

**Supplementary Table 1.** Overall injury types sustained by patients involved in MVAs in Qatar between 2016 and 2022.

**Type of injury (n = 1,473)**	**Number**	**Percentage (%)**
Blunt trauma	740	50.2
Fracture	351	23.8
Contusion	196	13.3
Abrasions	166	11.3
Crush injury	14	1.0
Hematoma	4	0.3
Penetrating injury	2	0.1

**Supplementary Table 2.** Initial Glasgow Coma Scale (GCS) of patients involved in Motor Vehicle Accidents in Qatar between 2016 and 2022 upon arrival to the Emergency Department.

**GCS**	**Number (n)**	**Percentage (%)**
3	24	1.6
5	3	0.2
6	1	0.1
7	1	0.1
8	1	0.1
9	2	0.1
10	1	0.1
11	2	0.1
12	2	0.1
13	6	0.4
14	7	0.5
15	1,428	96.6

**Supplementary Table 3.** Injury type in relation to age category for patients involved in MVAs in Qatar between 2016 and 2022.

	**Age group**		
	**<40 *n* (%)**	**≥40 *n* (%)**	***p***
Breathing/thoracic injury			
Yes	173 (13.0)	30 (21.1)	**0.007**
No	1160 (87.0)	112 (78.9)	
Orthopedic injury			
Yes	465 (34.9)	60 (42.3)	0.081
No	868 (65.1)	82 (57.7)	
Disability			
Yes	85 (6.4)	14 (9.9)	0.115
No	1248 (93.6)	128 (90.1)	

## Figures and Tables

**Table 1.** Demographic characteristics of desert motor vehicle accident patients included in the study.

	**Mean ± SD**	**Median (Q1–Q3)**
Age (*n* = 1,493)	24.6 ± 10.5	22 (17–31)
	Number (*n*)	Percentage (%)
Age (*n* = 1,493)		
<40	1,348	90.3
≥40	145	9.7
Gender (*n* = 1,496)		
Male	1,094	73.1
Female	402	26.9
Nationality (*n* = 1,474)		
Qatari	651	44.2
Resident	815	55.3
Visitor	8	0.5
Ethanol Intoxication (*n* = 1,478)
Yes	7	0.5
No	1,471	99.5
Pregnancy (*n* = 402)		
Yes	7	1.7
No	395	98.3

**Table 2.** Key characteristics related to the accidents that occurred in the desert in Qatar between 2016 and 2022.

**Type vehicle (*n* = 1,478)**	**Number (*n*)**	**Percentage (%)**
ATV	996	67.4
SUV	405	27.4
Motorcycle	75	5.1
Car	2	0.1
Protective gear (*n* = 1,478)
Yes	382	25.8
No	1,096	74.2
Mechanism injury (*n* = 1,480)
Collision	171	11.6
Rollover	970	65.5
Fall from vehicle	205	13.9
Others	134	9.1

ATV: all-terrain vehicle, SUV: sports utility vehicle.

**Table 3.** Outcome of patients involved in desert MVAs in Qatar between 2016 and 2022.

	**Number (*n*)**	**Percentage (%)**
Surgery (*n* = 1,476)		
Yes	267	18.1
No	1,209	81.9
Hospital length of stay (*n* = 1,493)	2.9 ± 7.3 (Mean ± SD)	1 (1–2) Median (Q1–Q3)
Alive (*n* = 1,496)		
Yes	1,474	98.5
No	22	1.5
Disability (*n* = 1,478)		
Yes	99	6.7
No	1,379	93.3
Outcome (*n* = 1,496)		
Stable	1,375	91.9
Disability	99	6.6
Death	22	1.5
Follow-up (*n* = 1,478)		
Yes	580	39.2
No	898	60.8

**Table 4.** Type of primary injuries sustained by patients involved in MVAs in Qatar between 2016 and 2022.

	**Number (*n*)**	**Percentage (%)**
Airway compromised (*n* = 1,496)	175	11.7
OMF trauma	120	8.0
Direct airway injury	40	2.7
Blood	19	1.3
Vomitus	13	0.9
Secretions	2	0.1
Foreign body	2	0.1
Others	9	0.6
Blunt neck trauma (*n* = 1,478)	134	9.1
C spine trauma (*n* = 1,478)	55	3.7
Fracture	49	3.3
Spinal cord injury	4	0.3
Dislocation	2	0.1
Thoracic trauma (*n* = 1,478)	206	13.9
Thorax fracture	83	5.6
Lung contusion	47	3.2
Thorax emphysema	25	1.7
Pneumothorax	22	1.5
Hemothorax	13	0.9
Flail chest	2	0.1
Cardiac/aortic trauma (*n* = 1,478)	26	1.8
Traumatic circulatory arrest	22	1.5
Blunt cardiac injury	3	0.2
Traumatic aortic dissection	2	0.1
Abdominal/pelvic trauma (*n* = 1,478)	122	8.3
Pelvic fracture	39	2.6
Solid organ injury	34	2.3
Fast positive	26	1.8
GU injuries	3	0.2
Pancreatic injuries	2	0.1
Hollow viscus injury	2	0.1
Duodenal injuries	1	0.1
Traumatic brain injury (*n* = 1,478)	223	15.1
Focal neurology	21	1.4
Skull fracture	14	0.9
ICH	11	0.7
Concussion	9	0.6
Axonal injury	7	0.5

OMF: oral and maxillofacial, GU: genitourinary, ICH: intracerebral hemorrhage.

**Table 5.** Types of secondary injuries sustained by patients involved in MVAs in Qatar between 2016 and 2022 by medical specialty.

	**Number (*n*)**	**Percentage (%)**
Orthopedic injuries (*n* = 1,478)	526	35.6
Closed fractures	331	22.4
Dislocation	59	4.0
Lumbosacral spine injury	40	2.7
Thoracic spine injury	36	2.4
Open fracture	31	2.1
Peripheral nerve injury	17	1.2
Degloving injury	13	0.9
Vascular injury	14	0.9
Tendon injury	4	0.3
Compartment syndrome	4	0.3
Rhabdomyolysis	2	0.1
OMF/ENT injuries (*n* = 1,478)	120	8.1
Nasal fracture	33	2.2
Lip/tongue injury	23	1.6
Mandibule injury	21	1.4
Nasal septal hematoma	14	0.9
Zygomatic injury	13	0.9
Auricular hematoma	13	0.9
Dental trauma	9	0.6
Lefort fracture	6	0.4
Tripod injury	1	0.1
Ophthalmology injuries (*n* = 1,478)	33	2.2
Orbital fracture	16	1.1
Hyphema	8	0.5
Conjunctival injury	7	0.5
Retrobulbar hematoma	6	0.4
Corneal injury	2	0.1
Ruptured globe	1	0.1
Ocular FB	1	0.1
Plastic surgery injuries (*n* = 1,478)	20	1.4
Friction burn	12	0.8
Amputation	4	0.3
Nailbed injury	1	0.1

OMF: oral and maxillofacial, ENT: ear/nose/throat.
